# Consequences of a warming climate for social organisation in sweat bees

**DOI:** 10.1007/s00265-016-2118-y

**Published:** 2016-04-30

**Authors:** Roger Schürch, Christopher Accleton, Jeremy Field

**Affiliations:** School of Life Sciences, University of Sussex, Brighton, BN1 9QG UK; CTU Bern, Department of Clinical Research, University of Bern, Bern, Switzerland; Institute of Social and Preventive Medicine (ISPM), University of Bern, Bern, Switzerland

**Keywords:** Sociality, Social polymorphism, Social plasticity, Climate change, Sweat bee, *Halictus rubicundus*

## Abstract

**Abstract:**

The progression from solitary living to caste-based sociality is commonly regarded as a major evolutionary transition. However, it has recently been shown that in some taxa, sociality may be plastic and dependent on local conditions. If sociality can be environmentally driven, the question arises as to how projected climate change will influence features of social organisation that were previously thought to be of macroevolutionary proportions. Depending on the time available in spring during which a foundress can produce worker offspring, the sweat bee *Halictus rubicundus* is either social or solitary. We analysed detailed foraging data in relation to climate change predictions for Great Britain to assess when and where switches from a solitary to social lifestyle may be expected. We demonstrate that worker numbers should increase throughout Great Britain under predicted climate change scenarios, and importantly, that sociality should appear in northern areas where it has never before been observed. This dramatic shift in social organisation due to climate change should lead to a bigger workforce being available for summer pollination and may contribute towards mitigating the current pollinator crisis.

**Significance Statement:**

The sweat bee *Halictus rubicundus* is socially polymorphic, expressing both solitary and social forms, and is socially plastic, capable of transitioning from solitary to social forms, depending on local environmental conditions. Here, we analyse detailed foraging data in relation to climate change predictions for Great Britain to show that worker numbers and sociality both increase under predicted climate change scenarios. Especially dramatic will be the appearance of social *H. rubicundus* nests in the north of Britain, where previously only solitary forms are found. Particularly, if more taxa are found to be socially plastic, environmentally driven shifts in social organisation may help to mitigate future pollinator crises by providing more individuals for pollination.

**Electronic supplementary material:**

The online version of this article (doi:10.1007/s00265-016-2118-y) contains supplementary material, which is available to authorized users.

## Introduction

In insects and vertebrates with parental care, a solitary life history entails a single female or breeding pair provisioning their own offspring. In contrast, eusociality is characterised by reproductive division of labour, where some members of the society forfeit their reproduction to help rear the offspring of others. The progression from solitary living to caste-based sociality is heralded as a major evolutionary transition (Maynard Smith and Szathmáry 1995). While it was originally believed that transitions between the two phenotypes must involve mutation and selection, social phenotype is now known to be influenced by both genetic and environmental factors (Schwander et al. 2010; Purcell 2011). For example, recent studies of both carrion crows and sweat bees have found that sociality may be plastic, with social phenotype being influenced by local environmental conditions (Baglione et al. 2002; Field et al. 2010; Field et al. 2012). Thus, while plasticity itself is likely to have a genetic basis, phenotypic differences between populations need not imply fixed genetic differences.

The social hymenopterans such as bees, wasps and ants that often live in large, complex societies are of tremendous ecological and economic importance (Aizen and Harder 2009; Ascunce et al. 2011; Kluser et al. 2011). The size of the social insect work force will often correlate with its level of ecological impact. For example, the pollination performance of a honey bee colony is affected by the number of colony workers (Free and Preece 1969), and the number of workers is often determined by ecological factors, such as the length of the foraging season (Bourke 1999). Changes in phenology, as a response to climate change, are documented in many animal groups (Parmesan 2006), but it is not known how warming temperatures will impact species whose colony size is causally dependent on environmental conditions.

**Fig. 1:**
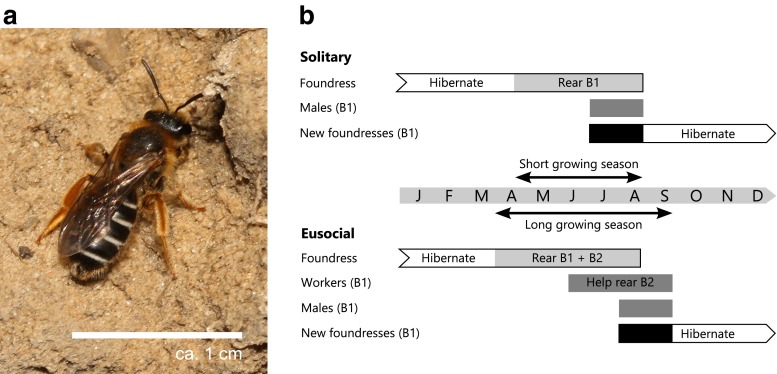
**a** *Halictus rubicundu*s nest foundress near her burrow entrance (photo credit: N. Owens). **b** Life cycle of *H. rubicundus* (after Chapuisat 2010). In solitary populations, the foundress rears one brood (B1) of both sexes. After mating, the female offspring hibernate and become foundresses the next year. In eusocial populations, the foundress raises a female biased B1 brood. Some of the B1 females enter hibernation (not shown in figure), but others assist the foundress in rearing a second brood consisting of both sexes (B2). Note that in social populations, provisioning activity lasts longer into autumn

The sweat bee *Halictus rubicundus* may be either social or solitary (i.e. socially polymorphic), depending on the length of the growing season (Eickwort et al. 1996; Soucy and Danforth 2002; Field et al. 2010; Soro et al. 2010). The following summary of the annual colony cycle is based on previous work (Yanega 1993; Hogendoorn and Leys 1997; Soucy 2002; Soucy and Danforth 2002; Chapuisat 2010; Field et al. 2010; Field et al. 2012). The cycle begins in spring, when each *H. rubicundus* foundress digs a separate nest burrow after leaving hibernation, where she alone rears a first brood (B1) of offspring (Fig. 1). In solitary populations (cooler climates), mated B1 females overwinter before re-starting the cycle the following spring, and there are no workers. In social populations (warmer climates), however, while some B1 females immediately overwinter, others become workers that forage to provision a second brood (B2) of overwintering offspring. Thus, in solitary populations, there is no pollen collecting after the spring foundress phase, whereas in social populations there is an additional summer phase with multiple bees (workers) provisioning each nest, providing a larger pollinator workforce at a time of year when there is negligible provisioning at solitary sites (foundresses very rarely provision in summer at solitary sites in the UK: J. Field & C. Bridge, unpubl data).

Using field transplant experiments, it was previously demonstrated that UK *H. rubicundus* foundresses are plastic in terms of the date in spring when they initiate provisioning for offspring, the size and maturation time of their offspring, their active daily provisioning time and the number of pollen loads that are collected daily. Critically, it was also demonstrated that plasticity in these individual behavioural traits leads overall to an induced switch between the two social phenotypes (social plasticity; Field et al. 2010; Field et al. 2012). However, no one has yet explored possible consequences of future environmental change on this process. If plasticity underlies the transition from solitary to social, which was previously believed to be a macroevolutionary process, and if this plasticity is environmentally driven, what may occur in the future in socially polymorphic bees such as *H. rubicundus*?

Here, we relate detailed foraging data for *H. rubicundus* to climate change predictions for Great Britain to assess when and where switches from solitary to social can be expected. The foundresses we observe in this study had been transplanted from Belfast, Northern Ireland to Lewes in southern England. Detailed behavioural observations over 4 years at the Belfast source site had shown that no workers at all are produced by the population nesting there (Supplementary Material of Field et al. 2010). In contrast, approximately half of the nests produced by Belfast foundresses transplanted to Lewes had workers, and only 25 % of nests had no summer provisioning at all. This change in social phenotype at Lewes must reflect plasticity and demonstrates the potential for a social phenotypic response to climatic warming that could occur in the absence of evolutionary change. Such a striking shift in social organisation due to climate change, from a solitary to a social phenotype, would lead to a bigger workforce being available for pollination in the summer months. Social polymorphism is widespread in sweat bees (Table S2), which represent one of the major bee lineages. Particularly, if other polymorphic species also turn out to be plastic, changes in social organisation and resultant increases in productivity could contribute towards mitigating the current pollinator crisis.

In this study, we will use observed relationships between temperature and foundress foraging and, in turn, between total spring foraging and number of workers to predict the number of workers that a nest would have under future temperature scenarios. Effectively, we will assume that female offspring produced before a certain time point will become workers. This is consistent with the general finding that, like other sweat bees, *H. rubicundus* is more likely to have social nests in locations with longer growing seasons (Soucy and Danforth 2002). It is also consistent with the specific finding that the nests in our dataset were more likely to have workers if the foundress began provisioning earlier in spring and produced B1 offspring earlier (Field et al. 2010). Similarly, Kocher et al. (2014) found that temperate zone, obligate social sweat bee species have a lower altitude distributional limit than solitary/socially polymorphic species, associated with longer foraging seasons lower down. Kocher et al. (2014) also suggested that interspecific differences in development time might interact with season length to determine species occurrence. Differences in development time between social and solitary sweat bees were small however (Kocher et al. 2014, Figure S4), and development time is often temperature dependent within species (Yanega 1997). In *H. rubicundus*, transplant experiments indeed show that development time is completely plastic, at least across the UK sites tested (Field et al. 2012).

Yanega (1989; 1997) proposed a more complicated mechanism that might determine caste and therefore social phenotype in *H. rubicundus*, known as the ‘mate limitation hypothesis’ (MLH). Under the MLH, females that mate within a short time of emergence as adults become physiologically competent to enter diapause and become foundresses the following year. In contrast, females that fail to mate within the time window become workers. Some workers can later become replacement queens, but not next year’s foundresses. The chance of mating, and hence the probability of entering diapause, might depend on the operational sex ratio (OSR) at the time of offspring emergence. Yanega (1989) presented correlational evidence in support of his hypothesis. For example, in his New York study population, the sex ratio of offspring in the mating pool appeared to become gradually more male-biased during the period when B1 offspring emerge as adults. In turn, the probability that a B1 female will become a worker is greater for early-emerging females than late emergers, until late in the season all females enter diapause rather than work. Thus, a decreasing probability of becoming a worker is indeed associated with an increasingly male-biased operational sex ratio. Under the MLH, foundresses might affect changes in social phenotype by adjusting the sex ratio of B1 offspring at the population level in response to environmental cues such as temperature (see Yanega 1989, 1997 for further discussion). However, the empirical data supporting Yanega’s hypothesis are only correlative. Lucas and Field (2013) investigated theoretically whether within-season patterns in the OSR observed by Yanega (1989) would be evolutionarily stable under the MLH. They found that the observed patterns could be stable, but under only quite restrictive conditions: when early-emerging workers are more valuable than late emergers in combination with some or all of the following: (i) high male mortality, (ii) low worker mortality and (iii) high worker productivity. Lucas and Field (2013) also noted that, under the MLH, B1 females do not choose strategically whether to become workers; it is simply a question of whether they happen to mate early in life. However, in situations where females would have greater inclusive fitness as workers than as hibernators, we might expect them to have evolved to become workers regardless of their mating status, and empirical data on the effect of mating on female caste in two other sweat bees are equivocal (Plateaux-Quénu and Packer 1998). Given the uncertainties about Yanega’s (1989, 1997) more complex hypothesis, we will assume that earlier emerging females become workers for strategic reasons, for example because there is more time left in the nesting season in which to be productive as a worker (Soucy and Danforth 2002; Field et al. 2010). Clearly, however, our conclusions about the short-term effects of climate change could be altered by future findings about the precise mechanism underlying caste determination.

## Methods

### Behavioural observations and temperature data

We made detailed observations of *H. rubicundus* nests from 22 April to 18 July 2009 in a garden in Lewes (50° 52′ 21″ N, 0° 0′ 32″ W). The foundresses were part of a transplant experiment (Field et al. 2010) and were moved to the site from a nesting aggregation in Belfast, Northern Ireland (54° 32′ 34″N, 5° 58′ 55″ W), mainly at the very start of the spring (some foundresses were transported the previous autumn; see Field et al. 2010 for more details). Each bee was individually marked with a pattern of enamel paint on the thorax, and the activity of the bee was monitored at the nest. Because nest entrances were within a few metres of each other, one observer (JF) could simultaneously monitor all nests. It was not possible to record data blind because our study involved focal animals in the field.

All bee entries to each nest with pollen were recorded continuously throughout almost everyday or part-day suitable for bee activity, with video recordings on some days used to verify data accuracy. Our interest focused on the foundress phase in spring (22 April to 31 May) when the single foundress in each nest provisions to produce the first (B1) offspring brood, potentially including workers (Field et al. 2010; Field et al. 2012). For this period, we extracted all provisioning events per day and per foundress. Observations from later in the year were used to establish the number of workers that each nest produced (Field et al. 2010).

To match behavioural observations of provisioning activity with temperature, we obtained data from a nearby weather station (IEASTSUS10, at Preston Park, Brighton) from www.wunderground.com. For each day of the focal spring period, we extracted the maximum temperature.

### Statistical analyses

We used R 3.0.2 for all statistical analyses (R Core Team 2013). For our first analysis, we calculated the proportion of actively provisioning nests, which we modelled as a response to maximum temperature with binomial errors and a logit-link function. We also modelled the number of provisioning events per day and per foundress as a function of temperature using Poisson errors with a log-link in a generalized linear mixed model using the package lme4 (Bates et al. 2013). Lastly, we analysed the number of workers per nest as a function of the total number of foundress provisioning trips using a generalised linear model with Poisson error. Because inspection of the relationship between temperature and foundress activity suggested quadratic relationships, we tested whether the addition of quadratic terms for temperature were significant improvements of model fits based on *χ*^2^ (binomial glm) and likelihood ratio tests (Poisson glmm; Faraway 2006). We inspected residuals and fitted values of final models to assess the validity of model assumptions (Faraway 2006).

### Data extraction from UKCP09

Given the relationship between temperature and foundress activity, on the one hand, and the relationship between foundress activity and the size of the worker force on the other, we wished to use simulated weather data to predict future worker numbers in *H. rubicundus*. These data are available for a variety of scenarios, ranging from a low emission scenario where humans manage to curb CO_2_ production, to a high emission scenario where we accelerate the use of fossil fuels.

We downloaded the boundaries of the regions of Great Britain from the Ordnance Survey OpenData web page (https://www.ordnancesurvey.co.uk/opendatadownload/products.html) as ESRI shape files (Table S1). We calculated the centroids of these regions using the packages ‘sp’ and ‘maptools’. For each of these 14 locations, we extracted simulated weather data for the different emission scenarios at different time frames using the Weather Generator 2.0 (© UK Climate Projections, 2009). We selected the standard weather generator data using a random sampling of the model variants (*N* = 100). We chose daily output and a run time of 30 years, which we stored as raw data. These data were then further processed in R to translate the daily maximum temperature into number of provisioning trips anticipated for an average foundress using our findings from the observational data. The number of provisioning trips during spring was then used to predict the number of worker offspring that foundresses will produce in the future.

For our predictions, we considered the time from the beginning of March to the end of May as the crucial period when foundresses can provision for worker offspring. Although provisioning in southern England today typically starts towards the end of April (Field et al. 2010; Field et al. 2012), we have observed it to begin in March in years with unusually warm early spring weather. In this study, we are concerned only with a potential first worker brood. If the whole season became longer under future climate change, the first worker brood could potentially help to produce an additional (second) worker brood before reproductives are produced, depending on what cues the emerging bees use to determine their own social status. Currently, we lack data on these cues, but Yanega has found that in Kansas, USA, *H. rubicundus* can produce two generations of workers (Yanega 1993). Here, we ignore plasticity in the number of generations per summer. Lastly, we incorporated foundress mortality by using the observed average lifespan of a foundress starting from the first provisioning date for any given simulated year (41.9 days).

## Results

### More foundresses are active—and are more active—when temperatures reach 20–21 °C

**Fig. 2:**
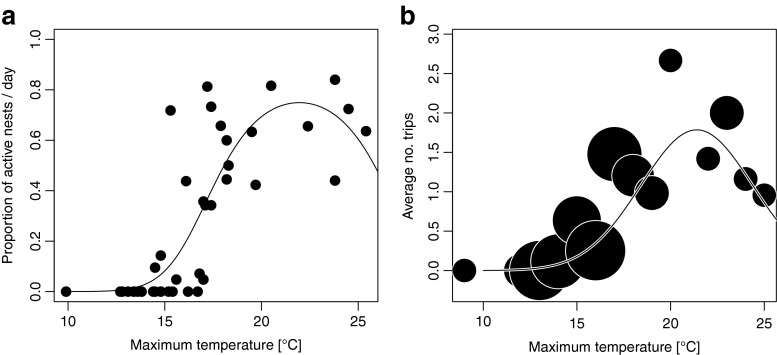
Activity of *H. rubicundus* in relation to temperature. **a** The proportion of active nests on a given day depended on the square of the maximum daily temperature so that most nests were active around 20 °C. See Table 1 for details of the fitted line. **b** The number of provisioning trips a foundress was able to complete depended on the squared maximum temperature on a given day so that the most trips could be performed around 20 °C. The size of the bubbles is proportional to the number of time a given 1 °C temperature interval was observed and contributed to the average depicted. The *fitted line* represents the activity of an average foundress while in reality each foundress had her own optimum. See Table 2 for details of the fitted line

In our study, foundresses were active on days where the maximum temperature reached between 9.9 and 25.4 °C, with a maximum proportion of foundresses active at 21 °C (Fig. 2a, Table 1). Similarly, foundresses on average made the maximum number of provisioning trips on days when temperatures were around 20 °C (Fig. 2b, Table 2).

**Table 1:** Parameter estimates from modelling the proportion of active nests on a given day as a function of the maximum temperature and the squared maximum temperature on that day. We used Binomial errors and a logit-link for our model

	Estimate	Std. error	*z*	*p*
	−33.842071	2.978942	−11.360	<0.001
Maximum temperature	3.182181	0.307565	10.346	<0.001
Maximum temperature^2^	−0.072463	0.007774	−9.321	<0.001

**Table 2:** Parameter estimates from modelling the number of trips a foundress makes on a day as a function of the maximum temperature and the squared maximum temperature on that day. We allowed for each female to have her own intercept and slope for the maximum temperature. Here, we used a Poisson error structure and a log-link to model the data. Temperature was scaled and centred for the analysis (centre: 16.81678, scale: 3.467088)

	Estimate	Std. error	*z*	*p*
	−26.0232	1.433536	−18.15	<0.001
Maximum temperature	2.531954	0.150975	16.77	<0.001
Maximum temperature^2^	−0.06213	0.003904	−15.92	<0.001

### Number of workers produced per nest depends on foundress spring provisioning activity

**Fig. 3:**
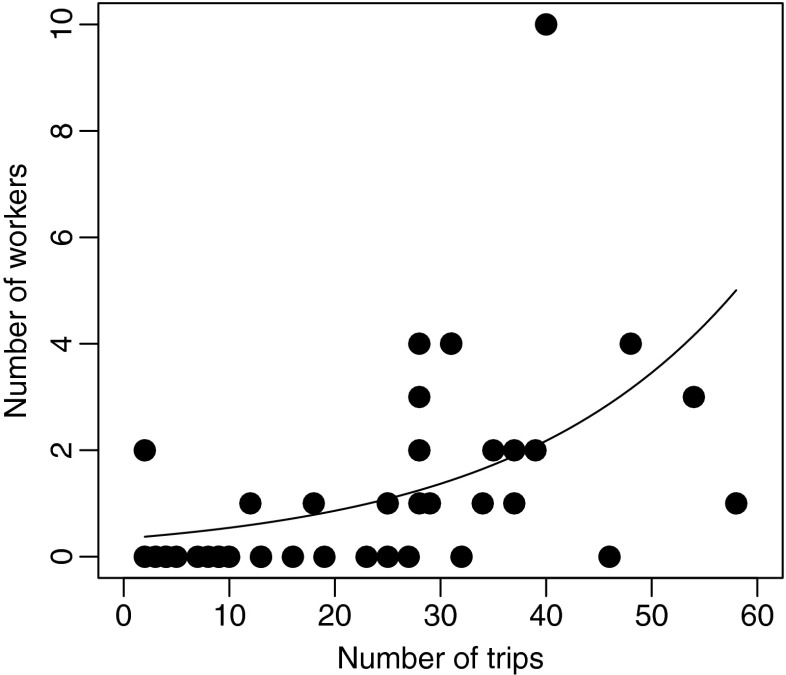
Worker numbers in relation to the number of spring provisioning trips. The more provisioning trips a female performed during spring time, the more worker offspring she would produce (see Table 3 for statistical details)

The number of workers produced at a particular nest (range 1–11) depended critically on the number of provisioning trips the foundress had undertaken during the spring time provisioning phase (Fig. 3, Table 3). The more provisioning trips a female performed during spring time, the more worker offspring she produced.

**Table 3:** Parameter estimates from modelling the number of worker offspring a foundress produced given the number of trips she made during the spring. Note that we modelled the number of workers as with Poisson errors and a log-link function

	Estimate	Std. Error	*z*	*p*
	−1.073650	0.344628	−3.115	0.001
Number of trips	0.046280	0.009232	5.013	<0.001

### For all future emission scenarios, bee worker numbers are projected to increase across Great Britain

**Fig. 4:**
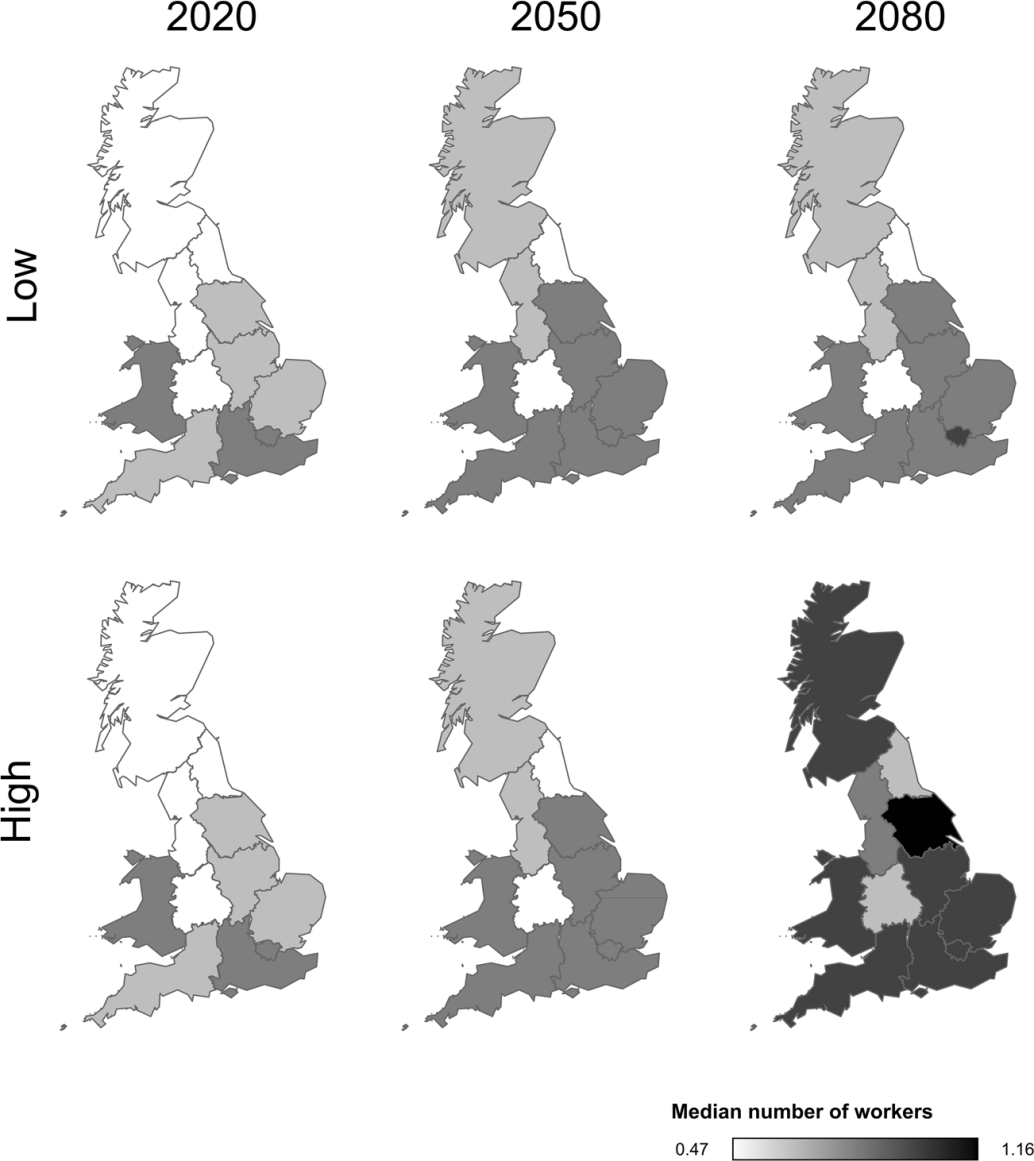
The effect of future climate change on worker numbers. For the centroid of each region of Great Britain (see Table S1), we have used weather predictions based on low and high CO_2_ emission scenarios to estimate the future number of workers an average foundress will be able to produce (see the main text for details on how we obtained the weather simulation data). Based on the worker numbers estimated for the centroid, regions were coloured from light (few workers) to dark (more workers)

For both low and high emission scenarios, we can expect an increase in worker numbers per foundress in *H. rubicundus* over vast areas of Great Britain in the next 70 years (Fig. 4), even in the absence of evolutionary change. The projected increase is most prominent in the southeast where only 50 % of nests are social today (median = 0.5 workers per nest (Field et al. 2010)). However, the most dramatic qualitative change is expected for northern bees, which are currently completely solitary.

## Discussion

Given that earlier emerging female offspring are more likely to become workers (Field et al. 2010), the number of *H. rubicundus* workers per nest should increase over Great Britain for both low and high emission climate scenarios. Foundresses will experience warmer temperatures in the springtime that, in turn, allow them to provision more and for more days. In this way, the projected increase in temperature should enable some foundresses at sites where all nests are currently solitary to have social nests. Human-induced climate change will thus lead to a switch in social organisation that is normally considered a major evolutionary transition. We stress that because the bees we observed were transplanted from a site in Belfast where nests are solitary, the worker production we observed at the destination site in Lewes is directly relevant to predicting phenotypic shifts in the absence of evolutionary change. We cannot compare the behaviour or fitness of these bees with bees native to the Lewes destination site because there was no native population present at Lewes. However, in another study where foundresses were transplanted from Belfast and a second northerly UK site to Sherborne in southern England, transplanted bees behaved very similarly to Sherborne natives in terms of date of nest initiation and foraging effort (Field et al. 2012). This suggests that *H. rubicundus* can respond efficiently to a new climate, and that little evolutionary change is needed to do so, at least across the range of conditions experienced at the sites concerned. If temperature increased more dramatically than in the scenarios we tested, foraging might eventually decrease without evolutionary change since we found that foraging effort was maximised at 20–21 °C (Fig. 2). Similarly, plants might also initially be more productive at higher temperatures, but productivity might decline if temperatures become too extreme (Hatfield and Prueger 2015).

Climate change may have other effects on sweat bee life strategies. For example, an increase in spring rainfall at sites where nests are currently social might cause foundresses to initiate provisioning later in the year so that there is no worker brood, with all B1 offspring instead entering hibernation (see Field et al. 2012, Fig. 4). On the other hand, an earlier start to spring with associated higher temperatures might enable a foundress in current solitary areas to suddenly produce workers. Indeed, origins of halictine sociality are associated with warmer environmental conditions (Brady et al. 2006; Field et al. 2010). Such changes could have economic consequences; gains or losses of sociality could mitigate—or accelerate—current pollinator losses that result from the intensification of farming and human developments (Potts et al. 2010; Kluser et al. 2011; Vanbergen et al. 2013).

Our findings possess wide applicability. *H. rubicundus* itself is a common bee with a Holarctic distribution. Climatically induced changes in social system could therefore potentially occur across the entire Northern hemisphere, although mitochondrial differentiation between solitary and caste-based populations in North America might indicate that plasticity is incomplete there (Soucy and Danforth 2002; Field et al. 2010). Furthermore, although plasticity has rarely been tested for directly, social polymorphism is widespread in sweat bees, one of the major bee lineages, having been demonstrated or inferred in at least 23 sweat bee species (Table S2) as well as in other bees (Michener 1990) and cooperatively breeding mammals (Vidya et al. 2009; Schradin et al. 2010) and birds (Komdeur 1992; Arnold and Owens 1999; Baglione et al. 2002). In the Seychelles warbler, for example, the extent to which individuals opt to live in families and help (‘work’) is dependent on available breeding habitat (Komdeur 1992). If climate change affects available habitats, cooperatively breeding birds could therefore quickly adapt to the new prevailing conditions. Carrion crows (*Corvus corone*) are known to adapt their social system quickly, depending on environmental conditions (Baglione et al. 2002). Similar rapid switches in social organisation have been observed in mammals: the striped mouse (*Rhabdomys pumilio*) alters social organisation, depending on population density, which is determined by climatic conditions (Schradin et al. 2010). Although not all socially polymorphic sweat bees are socially plastic (Plateaux-Quénu et al. 2000), the number of taxa known to have flexible social systems will only grow as more species are studied at multiple sites, over longer time periods and using common garden or transplant experiments.

The consequences of climate-induced shifts in sociality can be considered on two levels. First, there should be consequences for the population ecology and evolution of the focal species itself. Second, the way in which a focal species interacts with the environment will likely change, with implications for community and applied ecology. For temperate bees, the increase in number of workers per foundress should make nests more resilient to disturbance later in the year and ensure their survival when foundresses die (Eickwort et al. 1996; Field et al. 2010), leading to a greater proportion of nests surviving to produce foundresses for the following year (Sakagami and Fukuda 1989; Field et al. 2000). Additionally, higher productivity could result from more efficient task partitioning (Anderson and Ratnieks 1999; Grüter et al. 2013), as behavioural switching to sociality presumably occurs only when it leads to increased productivity. On the other hand, increased social conflict within groups could limit productivity: increased colony size can lead to increased ovarian development in first-generation worker offspring (Strohm and Bordon-Hauser 2003). In the longer run, a switch to sociality could induce further evolutionary changes. Once colonies become consistently larger, lower worker reproductive potential and higher morphological skew should coevolve (Bourke 1999). Indeed, sociality in some North American populations of *H. rubicundus* appears to be more specialised than in the UK, with greater morphological differentiation between queens and workers, and evidence of genetic differentiation between solitary and social forms (Soucy 2002; Soucy and Danforth 2002; Field et al. 2010). This probably reflects more extreme warm climates, where the solitary life cycle is rarely, if ever, expressed so that sociality is the only phenotype exposed to selection. By studying bees during the anticipated shift to sociality, we will be afforded a powerful opportunity to learn more about the role of social plasticity in the evolution of sociality (Crozier 1992; West-Eberhard 1992; West-Eberhard 2003), and the role of genetic accommodation in the evolution of a reproductive division of labour (Suzuki and Nijhout 2006).

An increased number of workers should increase the amount of pollination that bees provide (Free and Preece 1969). A larger number of workers could also mean that pollination services are more diverse, as individual bees often are flower constant (Heinrich 1976); more workers could presumably pollinate a greater diversity of flower species. Additionally, having more wild bees present during the summer and autumn months can increase the efficiency of sympatric honey bees as pollinators (Brittain et al. 2013). While such positive effects might occur, negative effects such as competitive exclusion could have detrimental effects on species that cannot respond to the warming climate as quickly (Inouye 1978; Sih 2013). Clearly, the implications of a switch from solitary to social organisation for ecological interactions require further study.

## Electronic supplementary material

Below is the link to the electronic supplementary material.

ESM 1 (DOCX 17 kb)
