# Supplementary material for: Consequences of a warming climate for social organisation in sweat bees
Source: Behav Ecol Sociobiol. 2016 Apr 30;70:1131–9. doi: 10.1007/s00265-016-2118-y (PMC4954839; doi:10.1007/s00265-016-2118-y)
Supplement: Supplementary file 1 — (DOCX 17 kb) [file 265_2016_2118_MOESM1_ESM.docx]

# Supplementary Material

**Table S1** The euro regions of Great Britain and their centroids in UK Grid coordinates. Altitudes were taken from Google Earth.

| Region | Easting | Northing | Altitude (AMSL) |
| --- | --- | --- | --- |
| Eastern | 573751.6 | 263775.9 | 81 |
| East Midlands | 481004.5 | 337236.9 | 44 |
| London | 531331.0 | 179645.8 | 7 |
| North West | 351884.3 | 461276.5 | 57 |
| Scotland | 276742.1 | 753732.2 | 466 |
| West Midlands | 381687.5 | 286831.3 | 117 |
| Yorkshire and the Humber | 451330.5 | 452107.7 | 15 |
| South East | 504235.6 | 155735.1 | 34 |
| North East | 406477.1 | 569468.4 | 123 |
| Wales | 281182.0 | 267809.0 | 433 |
| South West | 319702.7 | 123280.7 | 57 |

**Table S2** Exemplars of sweat bee species demonstrated or inferred to be socially polymorphic.

| Species | Representative references |
| --- | --- |
| HALICTINAE (SWEAT BEES) |  |
| GENUS *HALICTUS* |  |
| Halictus rubicundus | 1,14 |
| Halictus tumulorum | 8,15 |
| Halictus confusus | 8,15 |
| Halictus sexcinctus | 11 |
| GENUS *LASIOGLOSSUM* |  |
| SUBGENUS *LASIOGLOSSUM* |  |
| Lasioglossum mutilum | 4 |
| Lasioglossum scitulum | 5 |
| GENUS *LASIOGLOSSUM* |  |
| SUBGENUS *EVYLAEUS* |  |
| Lasioglossum calceatum | 12 |
| Lasioglossum albipes | 9 |
| Lasioglossum baleicum | 3 |
| Lasioglossum apristum | 6 |
| Lasioglossum fulvicorne | 10 |
| Lasioglossum boreale | 16 |
| Lasioglossum comagenense | 8,17 |
| Lasioglossum fratellum | 2 |
| Lasioglossum truncatus* | 1 |
| GENUS *LASIOGLOSSUM* |  |
| SUBGENUS *DIALICTUS* |  |
| Lasioglossum problematicum | 8 |
| Lasioglossum pilosum* | 1 |
| Lasioglossum rohweri* | 1 |
| Lasioglossum pacatus | 1 |
| Lasioglossum laevissimum* | 1 |
| Lasioglossum nigroviridis* | 1 |
| GENUS *AUGOCHLORELLA* |  |
| Augochlorella striata | 7,17 |
| GENUS *MEGALOPTA* |  |
| Megalopta genalis | 13 |

‘Socially polymorphic’ is here taken to mean that some nests exhibit a high reproductive skew at one or more stages of the life-cycle, normally associated with a division of labour between foragers and a non-foraging reproductive; while other nests always have just one female which both oviposits and forages alone.

*Species inferred as polymorphic because it is known to exhibit sociality in some populations but also occurs in boreal regions where the growing season is too short to permit more than one annual brood

**References for Table S21**

1. Eickwort, G.C., Eickwort, J.M., Gordon, J. & Eickwort, M.A. Solitary behavior in a high altitude population of the social sweat bee *Halictus rubicundus* (Hymenoptera: Halictidae). *Behav. Ecol. Sociobiol.* **38**, 227-233 (1996).

2. Field, J. Patterns of provisioning and iteroparity in a solitary halictine bee, *Lasioglossum* (*Evylaeus*) *fratellum* (Perez), with notes on *L* (*E*) *calceatum* (Scop) and *L* (*E*) *villosulum* (K). *Insectes Soc.* **43**, 167-182 (1996).

3. Hirata, M. & Higashi, S. Degree-day accumulation controlling allopatric and sympatric variations in the sociality of sweat bees, *Lasioglossum* (*Evylaeus*) *baleicum* (Hymenoptera : Halictidae). *Behav. Ecol. Sociobiol.* **62**, 1239-1247 (2008).

4. Miyanaga, R., Maeta, Y., Mizuta, G. Discovery of social nests in a bivoltine, basically solitary halictine bee, *Lasioglossum* (*Lasioglossum*) *mutilum* (Vachal) (Hymenoptera: Halictidae). *Entomol. Sci.* **1**, 165-169 (1998).

5. Miyanaga, R., Maeta, Y., Hoshikawa, K. Nesting biology and occurrence of social nests in a bivoltine and basically solitary halictine bee, *Lasioglossum* (*Lasioglossum*) *scitulum* Smith (Hymenoptera: Halictidae). *Entomol. Sci.* **3**, 291-302 (2000).

6. Miyanaga, R., Maeta, Y. & Sakagami, S.F. Geographical variation of sociality and size-linked color patterns in *Lasioglossum (Evylaeus) apristum* (Vachal) in Japan (Hymenoptera, Halictidae). *Insectes Soc.* **46**, 224-232 (1999).

7. Packer, L. Solitary and Eusocial nests in a population of *Augochlorella striata* (Provancher) (Hymenoptera, Halictidae) at the Northern edge of Its range. *Behav. Ecol. Sociobiol.* **27**, 339-344 (1990).

8. Packer, L. The relevance of phylogenetic systematics to biology: examples from medicine and behavioral ecology. *Mem. Mus. natn. Hist. nat.* **173**, 11-29 (1997).

9. Plateaux-Quenu, C. Flexibilite sociale chez *Evylaeus albipes* (F.) (Hymenoptera, Halictinae). *Actes des Colloques des Insectes Sociaux* **8**, 127-134 (1993).

10. Plateaux-Quenu, C. Subsociality in halictine bees. *Insectes Soc.* **55**, 335-346 (2008).

11. Richards, M.H. Variable worker behaviour in the weakly eusocial sweat bee, *Halictus sexcinctus* Fabricius. *Insectes Soc.* **50**, 361-364 (2003).

12. Sakagami, S.F., Munakata, M. Distribution and bionomics of a transpalearctic eusocial halicine bee, *Lasioglossum* (*Evylaeus*) *calceatum*, in northern Japan, with reference to its life cycle at high altitude. *Journal of the Faculty of Science of Hokkaido University (Series VI, Zoology)* **18**, 411-439 (1972).

13. Smith, A.R., Kapheim, K.M., O'Donnell, S. & Wcislo, W.T. Social competition but not subfertility leads to a division of labour in the facultatively social sweat bee *Megalopta genalis* (Hymenoptera: Halictidae). *Anim. Behav.* **78**, 1043-1050 (2009).

14. Field, J., Paxton, R. J., Soro, A. & Bridge, C. Cryptic plasticity underlies a major evolutionary transition. *Curr Biol* **20**, 2028–2031 (2010).

15. Danforth, B.N., Sauquet, H. & Packer, L. Phylogeny of the bee genus *Halictus* (Hymenoptera : Halictidae) based on parsimony and likelihood analyses of nuclear EF-1 alpha sequence data. *Mol. Phylogenet. Evol.* **13**, 605-618 (1999).

16. Danforth, B.N., Conway, L. & Ji, S.Q. Phylogeny of eusocial *Lasioglossum* reveals multiple losses of eusociality within a primitively eusocial clade of bees (Hymenoptera : Halictidae). *Syst. Biol.* **52**, 23-36 (2003).

17. Packer, L., Jessome, V., Lockerbie, C. & Sampson, B. The phenology and social biology of four sweat bees in a marginal environment - Cape-Breton-Island. *Can. J. Zool.* **67**, 2871-2877 (1989).
